# A Practical Treatise on the Diseases of Children

**Published:** 1859-05

**Authors:** 


ART. VII.—*A Practical Treatise on the Diseases of Children.* By D. FRANCIS CONDIE, M.D., etc. Fifth edition, revised and enlarged. 8vo., pp. 762. Philadelphia: Blanchard & Lea, 1858.

WE should deem it an act of supererogation to do more than notice the excellent work of Dr. Condie, which first appeared fifteen years ago, and is now presented to us as the fifth edition; sufficient commendation for its high worth and the position which it assumed from the very commencement as a standard work upon pædiatrics. The author has indeed written a "practical treatise," and the young student, as well as the practitioner, will find that every subject has been discussed with the accustomed clearness and fullness of the author. We congratulate Dr. Condie upon the success of his work, which must be classed among the very best in any language.

